# Barriers to Real-Time Medical Direction via Cellular Communication for Prehospital Emergency Care Providers in Gujarat, India

**DOI:** 10.7759/cureus.676

**Published:** 2016-07-08

**Authors:** Benjamin Lindquist, Matthew C Strehlow, G.V. Ramana Rao, Jennifer A Newberry

**Affiliations:** 1 Department of Emergency Medicine, Stanford University School of Medicine; 2 Emergency Medicine Learning Centre (EMLC) & Research, GVK Emergency Management and Research Institute

**Keywords:** prehospital emergency care, ems, global health, international medicine

## Abstract

Background: Many low- and middle-income countries depend on emergency medical technicians (EMTs), nurses, midwives, and layperson community health workers with limited training to provide a majority of emergency medical, trauma, and obstetric care in the prehospital setting. To improve timely patient care and expand provider scope of practice, nations leverage cellular phones and call centers for real-time online medical direction. However, there exist several barriers to adequate communication that impact the provision of emergency care. We sought to identify obstacles in the cellular communication process among GVK Emergency Management and Research Institute (GVK EMRI) EMTs in Gujarat, India.

Methods: A convenience sample of practicing EMTs in Gujarat, India were surveyed regarding the barriers to call initiation and completion.

Results: 108 EMTs completed the survey. Overall, ninety-seven (89.8%) EMTs responded that the most common reason they did not initiate a call with the call center physician was insufficient time. Forty-six (42%) EMTs reported that they were unable to call the physician one or more times during a typical workweek (approximately 5-6 twelve-hour shifts/week) due to their hands being occupied performing direct patient care. Fifty-eight (54%) EMTs reported that they were unable to reach the call center physician, despite attempts, at least once a week.

Conclusion: This study identified multiple barriers to communication, including insufficient time to call for advice and inability to reach call center physicians. Identification of simple interventions and best practices may improve communication and ensure timely and appropriate prehospital care.

## Introduction

Emergency medical care is receiving growing attention in low- and middle-income countries (LMICs) as acute conditions, such as road traffic accidents, continue to rise as leading causes of mortality [1]. Several models for prehospital care systems in LMICs exist with varying success. Most LMICs, especially in rural settings, lack a centralized and coordinated prehospital system and rather depend on untrained laypersons, commercial drivers, or volunteers to transport patients to healthcare facilities [2]. Some countries have trained a cadre of emergency medical technicians (EMTs), while others have focused on educating community health workers (CHWs) [3-5]. In some countries such as India, cellular phones are used to provide real-time physician medical direction to the prehospital care providers and thus improve the quality of prehospital emergency care [3-5]. In the United States (US) and other nations with robust prehospital care systems, some suggest that online medical direction for prehospital services may be unnecessary and time-consuming while others purport improved clinical outcomes [6-7]. However, recommendations for emphasizing standing orders over online medical direction are based on US studies where paramedics have significantly more training than EMTs or other prehospital healthcare providers operating in most LMICs.

Cellular communication is particularly beneficial in supporting emergency services in rural areas [8]. Globally, no robust clinical trials have evaluated the benefit of cellular communication in the prehospital environment, but several small studies have shown promising results in improving patient care in other clinical arenas [8-10]. For example, LMIC community health workers (CHWs) and midwives have been given cellular devices to enable real-time medical direction from physicians for the management of emergent conditions, particularly maternal and neonatal emergencies [4, 8].

In India, the public-private partnership GVK Emergency Management and Research Institute (GVK EMRI) provides prehospital emergency medical care for a catchment area spanning 17 states and union territories, covering nearly 750 million people [4]. To reach this large population, GVK EMRI trains EMTs to address abnormalities in circulation, airway, and breathing. EMTs take a 52-day foundational course on basic prehospital emergency care such as CPR in cardiac arrest, management of massive hemorrhage, basic airway support with jaw thrust or chin lift maneuvers, and breathing support with bag mask ventilation. Currently, GVK EMRI employs 19,546 EMTs, with 14,855 (76%) staffing emergency care ambulances, 4,496 (23%) providing specific maternal transport services, and 195 (1%) working in an emergency care centers. Of these EMTs, 390 (2%) have received an extended two-week training in advanced emergency care, which includes advanced airway management, ventilator management, and interpretation of electrocardiograms. Collectively, EMTs manage approximately 28,000 patients/day. The average EMT cares for 14 patients/week, receiving online medical direction in approximately 4.6 patients/week.

In India, EMTs rely on 24/7 call center physician supervision to provide the majority of emergency care and expand their practice to more advanced emergency care, including intravenous medication administration. Call center physicians support EMTs in following established protocols and provide additional medical direction, based on pre-established physician-specific protocols. The protocols explicitly state which treatments the EMTs are able to complete via standing orders and which actions require physician direction (Appendix B).

In the majority of Indian states covered by GVK EMRI services, EMTs are not required to call physicians for every patient (e.g. interfacility transport or minor orthopedic injury), but must receive physician guidance in managing all cases considered critical, as defined by GVK EMRI. Critical cases comprise approximately 9,240 (33%) of all patients seen daily. For the proper functioning of this system, communication between healthcare providers must be efficient and reliable. We sought to evaluate and identify barriers to effective cellular communication in a convenience sample of GVK EMRI EMTs in the state of Gujarat, India.

## Materials and methods

This was a cross-sectional study of practicing GVK EMRI EMTs in Gujarat, India. GVK EMRI has provided emergency medical services in Gujarat for eight years and now manages a fleet of 585 ambulances to provide care across the state. A single, central call center in the city of Ahmedabad manages all calls, ambulance dispatch, and physician online medical direction. In the state of Gujarat, in contrast to the majority of states where GVK EMRI operates, EMTs are required by the state level leadership to call physicians for every patient encounter, regardless of severity. Cellular network strength is considered strong in Gujarat [11]. Further, to increase the probability that a particular EMT’s catchment area has sufficient cellular coverage, each EMT carries two subscriber identification module (SIM) cards from different network providers. Research assistants in Gujarat surveyed a convenience sample of practicing EMTs during a two-day period in February 2016. In that month, 58,286 prehospital cases received online medical direction from call center physicians with an average of 1,982 patients/day. Individual physicians handled an average of 198 cases/day.  

The survey was designed to capture two different processes: 1) to identify why calls were not initiated, and 2) once the call was initiated, why calls could not be completed. (Appendix A). Any practicing EMT on duty was eligible for the study. EMTs were excluded if they were with a patient at the time of the research assistant’s call. EMTs provided verbal consent to participate in the survey. Research assistants contacted EMTs by phone and collected data using a structured questionnaire. All research assistants are trilingual and conducted all surveys in the language most comfortable for the EMT, whether Hindi, Gujarati, or English. No identifying information was collected, and the authors were blinded as to which EMTs completed the survey. We analyzed the data using descriptive statistics, including absolute frequencies, means, and standard deviations. Ethical approval was obtained for this study from both GVK EMRI and the Institutional Review Board from Stanford University (IRB # 37656).

## Results

Research assistants collected a total sample of 108 EMTs (9.1%), out of 1180 total EMTs in Gujarat, with 100% response rate and no refusals to participate. Demographic information is included in Table 1. Mean age was twenty-six years (SD 4.1), and mean experience was four years (SD 2). EMTs worked an average of 5.6 twelve-hour shifts/week. 

**Table 1:** Respondent Demographics

	*n*	%
Gender		
Male	73	67.6
Female	35	32.4
Age (years)		
20-24	45	41.7
25-30	45	41.7
>30	18	16.6
Experience (years)		
< 1	1	0.9
1-2	34	31.5
3-4	42	38.9
> 4	31	28.7
Calls to physician/week		
< 10	3	2.8
10-19	58	53.7
20-29	35	32.4
> 30	12	11.1

**Figure 1:**
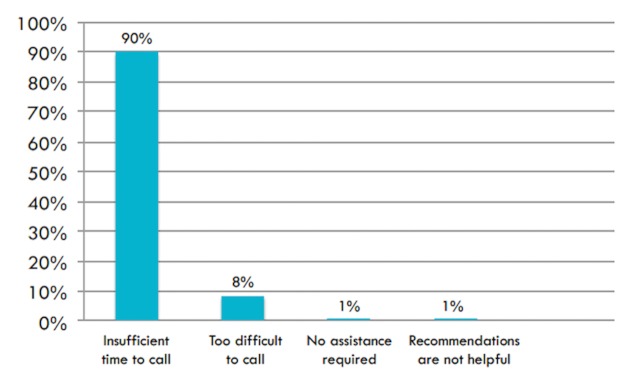
Most Common Reasons EMTs Do Not Initiate Calls for Medical Direction in Gujarat, India

Overall, ninety-seven (89.8%) EMTs responded that the most common reason they did not initiate a call with the call center physician was because of insufficient time (Figure 1). 

**Figure 2:**
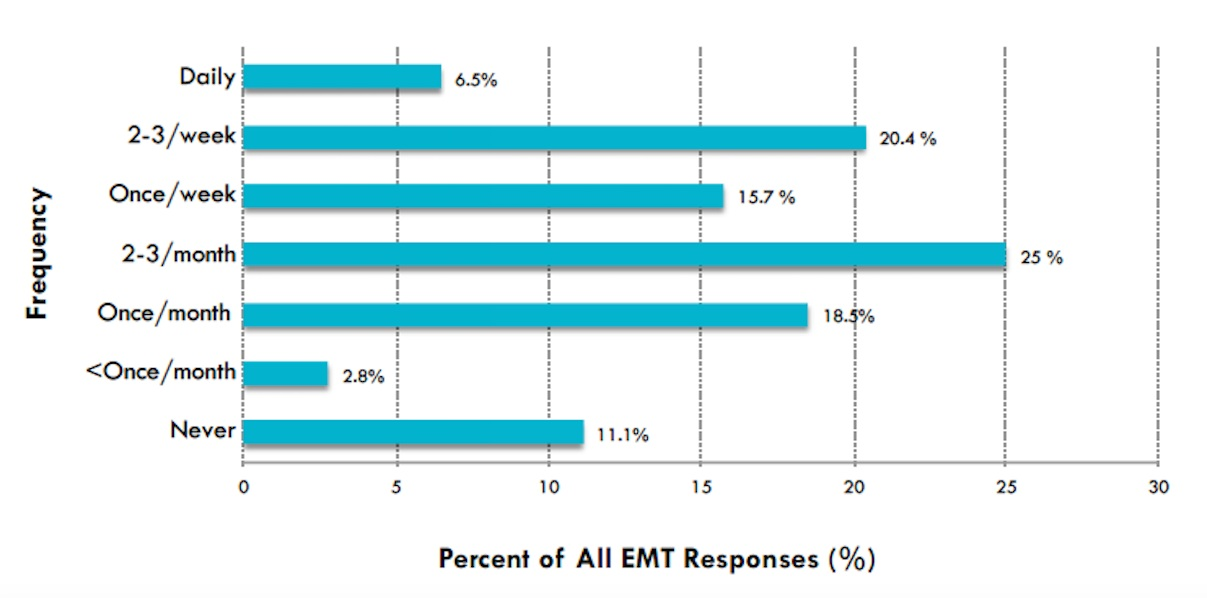
EMTs' Reported Frequency of Not Calling for Medical Direction Due to Patient Care Needs

In assessing phone call initiation by EMTs, forty-six (42%) EMTs reported that they were unable to initiate a call to the physician one or more times during a typical workweek due to their hands being occupied performing direct patient care (Figure 2). Of the 46 EMTs who reported they were unable to call the call center physician one or more times a week due to their hands being occupied, thirty-nine (85%) reported that the reason was insufficient time, and seven (16%) reported it was too difficult to call. 

In evaluating phone call connection, fifty-eight (54%) EMTs reported they were unable to reach the call center physician, despite attempts, one or more times each week. Of those 58 EMTs, nineteen (32.8%) reported they were unable to reach the call center physician because the call was dropped, while four (6.8%) reported no one was available to answer the call. EMTs most commonly selected ‘other’ (n = 35; 60.3%) as the reason the phone call connection failed, but did not provide further explanation. Finally, in evaluating barriers to completion of medical direction, thirty-eight (35%) EMTs reported that at least once a month they were unable to finish the call with the physician, and receive final recommendations, once initiated due to the patient requiring assistance. 

## Discussion

Prehospital care in LMICs is receiving increased attention due to growing mortality from acute conditions [1]. While there is no perfect system that fits all LMICs, several models exist for the proper functioning of prehospital care in low resource countries [2]. One such model uses cellular communication to expand the scope of EMTs and CHWs to manage emergencies under the direction of physicians [4]. However, timely communication is imperative for such programs to work effectively.

Our study showed that barriers exist to call initiation and completion in a small sample of EMTs in a robust prehospital system in India. Specifically, EMTs are often occupied with direct patient care and, therefore, unable to communicate with physicians in the call center in a timely fashion. This delay may lead to lapses in appropriate, protocol-directed care for emergency patients.

This study has certain limitations. The self-reported survey data is subject to recall bias. Almost 27% of EMTs reported not calling for medical direction due to patient needs at least 2-3 times per week. However, according to call center data, respondent EMTs averaged 19.5 patients/week (SD 9.6) with 19 calls to the physician/week (SD 7.4) during the month of February. Notably, there are wide standard deviations in this data, which may account for the distribution of responses seen in this study. Furthermore, sampling bias may be present since the population studied was a convenience sample of EMTs working on a single day. Similarly, undercoverage bias is likely since the survey was conducted during the day and did not include EMTs working at night who may have unique challenges.

However, the results are still informative and will help direct future studies that evaluate prehospital communication processes more thoroughly. Many states do not have as high of rates of calling for medical direction. Rather, the national average is 4.6 patients/week receiving medical direction out of an average 14 patients/week seen. Consequently, the barriers identified here may have an even greater impact in other states.

Future investigations should evaluate the effectiveness of hands-free devices in improving cellular phone communication. Other studies should identify types of patient complaints and times of day where cellular communication is ineffective in order to target specific improvement interventions. Finally, future research should identify whether failure to call the physician for online medical direction is correlated with worse clinical outcomes. 

## Conclusions

Cellular phones and other forms of telemedicine have enormous potential to expand the scope of prehospital care services in LMICs where the rates of traumatic illnesses and other emergent medical conditions continue to rise. However, existing barriers prevent effective real-time communication. Best practices, which may include the use of hands-free technology, need to be established. In India, further work is needed to improve phone communication as a means of supporting more comprehensive prehospital care. 

## Figures and Tables

**Figure 3:**
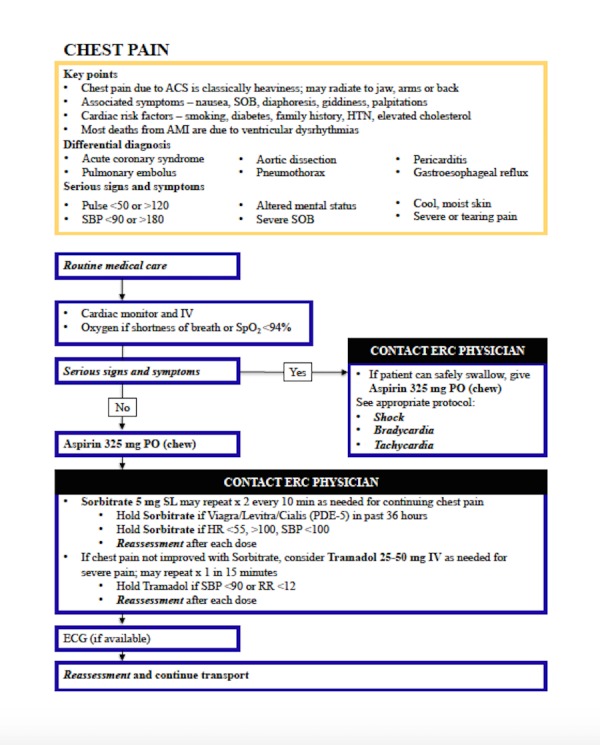
Chest Pain Protocol I

**Figure 4:**
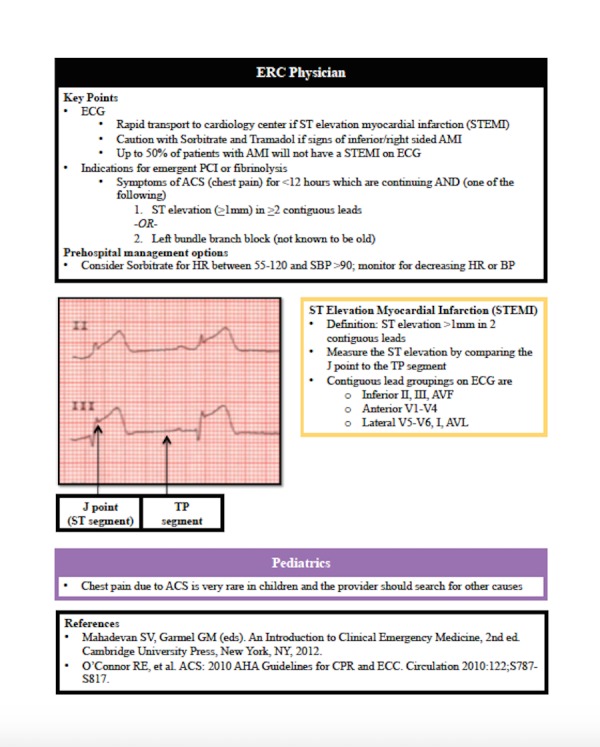
Chest Pain Protocol II

## Appendices

Appendix A: Cellular Communication Survey

What is the most common reason you do NOT call the ERC physician? (select one)

1. There is not enough time to complete the phone call
2. The ERC physician recommendations are not helpful
3. It’s too difficult to speak on the phone while treating the patient
4. You do not require assistance

How often are you unable to call the physician because your hands are occupied caring for the patient?

1. Never
2. Less than once a month
3. Once a month
4. 2-3 times a month
5. Once a week
6. 2-3 times a week
7. Daily

How often do you stop the call with the ERC physician before you want to be finished because the patient is ill and requires your assistance?

1. Never
2. Less than once a month
3. Once a month
4. 2-3 times a month
5. Once a week
6. 2-3 times a week
7. Daily

When calling to speak with the ERC physician, how often are you unable to reach them?

1. Never
2. Less than once a month
3. Once a month
4. 2-3 times a month
5. Once a week
6. 2-3 times a week
7. Daily

When you try to call the ERC physician, what is the most common reason you are unable to speak with him or her?

1. No one is available
2. Call is dropped
3. Other

Appendix B: Chest Pain Protocols
